# circRNADb: A comprehensive database for human circular RNAs with protein-coding annotations

**DOI:** 10.1038/srep34985

**Published:** 2016-10-11

**Authors:** Xiaoping Chen, Ping Han, Tao Zhou, Xuejiang Guo, Xiaofeng Song, Yan Li

**Affiliations:** 1 Department of Biomedical Engineering, Nanjing University of Aeronautics and Astronautics, Nanjing 211106, China; 2 Department of Gynecology and Obstetrics, The First Affiliated Hospital with Nanjing Medical University, Nanjing 210029, China; 3 State Key Laboratory of Reproductive Medicine, Department of Histology and Embryology, Nanjing Medical University, Nanjing 210029, China; 4 Center of Pathology and Clinical Laboratory, Sir Run Run Hospital Affiliated with Nanjing Medical University, Nanjing 211166, China

## Abstract

It has been known that circular RNAs are widely expressed in human tissues and cells, and play important regulatory roles in physiological or pathological processes. However, there is lack of comprehensively annotated human circular RNAs database. In this study we established a circRNA database, named as circRNADb, containing 32,914 human exonic circRNAs carefully selected from diversified sources. The detailed information of the circRNA, including genomic information, exon splicing, genome sequence, internal ribosome entry site (IRES), open reading frame (ORF) and references were provided in circRNADb. In addition, circRNAs were found to be able to encode proteins, which have not been reported in any species. 16328 circRNAs were annotated to have ORF longer than 100 amino acids, of which 7170 have IRES elements. 46 circRNAs from 37 genes were found to have their corresponding proteins expressed according mass spectrometry. The database provides the function of data search, browse, download, submit and feedback for the user to study particular circular RNA of interest and update the database continually. circRNADb will be built to be a biological information platform for circRNA molecules and related biological functions in the future. The database can be freely available through the web server at http://reprod.njmu.edu.cn/circrnadb.

Unlike linear RNA, circular RNA is a special group of non-coding RNA which forms a covalently closed continuous loop from exon circularization. In classical molecular biology, precursor RNA produced from DNA template strand by transcription can be processed into mature linear messenger RNA by canonical RNA splicing, in which introns are removed, while exons connect together in genomic order. However, non-canonical splicing can make exons scrambled to form a circle^1^,^2^. The first circular RNA was recognized in the 1970s. In 1979, the researcher suggested that RNAs could exist in circular form in the cytoplasm of eukaryotic cells^3^. Ten years later, it was reported that human cytoplasmic RNA contained very low levels of transcripts of the DCC gene with scrambled exons^4^. For the next few decades, due to the specificity of the structure and the low expression level of circRNA, only a few genes were identified to express circRNAs, including DCC, EST-1, SRY etc. Recently, with the development of high throughput sequencing technology, a large number of circRNAs has been discovered across species^5^,^6^,^7^,^8^. These circRNA molecules were found to be evolutionary conservative, stable, and specifically expressed across tissues or developmental stages^9^,^10^,^11^,^12^. It has been shown that they play important roles in gene regulation^9^,^13^. Therefore circular RNA has become the hotspots in the current transcriptomics research field.

Recently, as researchers put a lot of efforts into the study of circRNA, building a comprehensive circular RNA database become imperative. Several databases of the circRNA have been published, such as circBase, circRNABase and Circ2Trait^14^,^15^,^16^. The circBase merged and unified data sets of circRNAs from public references, with the evidence supporting their expression within the genomic context^14^. Circ2Traits is a comprehensive database for circRNA potentially associated with disease and traits^15^, which has only 1954 circRNAs. circRNABase is designed for decoding miRNA-circRNA interaction networks from thousands of circRNAs and 108 CLIP-Seq (HITS-CLIP, PAR-CLIP, iCLIP, CLASH) datasets^16^, however it does not provide the genomic information of circRNAs.

In order to further study the circRNA and related biological functions, we build a comprehensive reference database, named as circRNADb. We collected dataset of circRNAs from relevant literatures, together with the circRNAs dataset identified from the Gliomas RNA-Seq dataset by our research group^17^. However, the primary data may have false positives (circRNAs with two ends from different genes) and redundancy, so we filtered the dataset according to gene annotation GTF file, and obtained a total of 32,914 human exonic circRNAs. Its detailed genomic information are also listed in the database, including its best matched transcript and the corresponding exon splicing information, genome sequences, in addition to all the possible isoforms and the corresponding exon splicing information.

Although circRNA is classified as a non-coding RNA, researchers have reported that eukaryotic ribosome can initiate translation on circRNA, but only when the RNA contains internal ribosome entry site (or IRES) elements^18^. In 1995, Chen and colleagues showed that a synthetic circRNA containing IRES elements could recruit the ribosome to initiate translation, whereas those circRNAs without IRES did not^18^. Although the tested circRNA was a purely artificial construct, Chen and colleagues stated in their paper that they would be interested to see whether natural circRNAs contain IRES elements. So in this work we annotated the internal ribosome entry site and open reading frame (ORF) for the circRNA with protein-coding potential. Their protein expression evidences by mass spectrometry were also provided. Besides, we analyzed the features of the proteins translated from circRNAs, included domains, N-Glycosylation sites, mucin type O-Glycosylation sites and phosphorylation sites. Users can also employ “SProtP Human” to recognize those short-lived proteins based on sequence-derived features^19^.

Finally, human circRNA data sets, along with its genomic features, protein coding potential and protein features were integrated into circRNADb.

## Materials and Methods

Raw circRNAs dataset in circRNADb were collected from related literatures^9^,^10^,^11^,^12^,^17^. We filtered those circRNAs supported by only one read, and only included those circRNAs supported by two or more reads. Basic genomic information about the circRNAs from the above dataset was extracted in BED format, including chromosome name, start position, end position and the cell (or tissue) type. Aided by gene annotation GTF file, we obtained the circRNA genomic information, such as strand, gene symbol, all possible transcription and the corresponding exon splicing information. The longest possible spliced transcript was taken as the best candidate sequence of the circRNAs.

It has been known that canonical ribosome-based translation needs 5′-cap structure^18^. The alternative mechanism to initiate translation in eukaryotic mRNAs is that the RNA has internal ribosome entry site^18^. As circRNAs don’t have 5′-cap structure, another structure used for ribosome entry is internal ribosome entry site. An IRES element is a nucleotide sequence that allows the ribosome initiate translation directly in the middle of the mRNA sequence, instead of reading from the 5′ head to 3′ end^20^. If a circRNA contains at least one IRES element, it may be able to encode a protein. Thus, in order to annotate the protein-coding potential of all the circRNAs, we employed the method of VIPS depending on RNA structure similarity as proposed by Hong J. J. *et al*. to predict the IRES element in the spliced sequence of each circRNA^21^. VIPS has three key steps: RNA folding, RNA secondary structure comparison and pseudoknot prediction program^21^. RNALfold (from ViennaRNA package, version 2.1.9), RNA Align (provided by corresponding author of VIPS) and pknotsRG (version 1.3) were used in the IRES prediction system. RNALfold was used to predict local stable RNA secondary structures of long RNA by minimum free energy method^22^, while pknotsRG employed the newest Turner energy rules for finding the structure of minimal free energy, so it was able to predict a restricted class of pseudoknots^23^.

In addition to IRES elements, the longest open reading frame was predicted for each circRNA. Any frame that starts with a start codon, and ends with a stop codon with a length longer than 300 bp were considered as an ORF for protein coding. Furthermore, features of the proteins translated from circRNAs were analyzed. SMART was used to detect domains. NetNGlyc 1.0, NetOGlyc 3.1 and NetPhos 3.1 were employed to predict N-Glycosylation sites, Mucin type GalNAc O-Glycosylatiokn sites and phosphorylation sites respectively^24^,^25^,^26^,^27^,^28^. “SProtP Human” was also used to distinguish those short-lived proteins^19^.

To verify the potential coding ability of circRNA, two public proteomic datasets were used to search for the existence of fragmental peptides encoded by circRNAs. The raw data of human brains were downloaded from the PRoteomics IDEntifications (PRIDE) database (data identifies: PXD000458 and PXD002528)^29^. The parameters including quantification and modifications for database search were the same as the original studies. However, to obtain reliable identification results, an integrated database of the whole human protein sequences (UniProt release: 2013_07; 133806 sequences) and the predicted protein sequences exclusively encoded by circRNAs was applied to identify unique peptides encoded by circRNAs.

**Figure 1:**
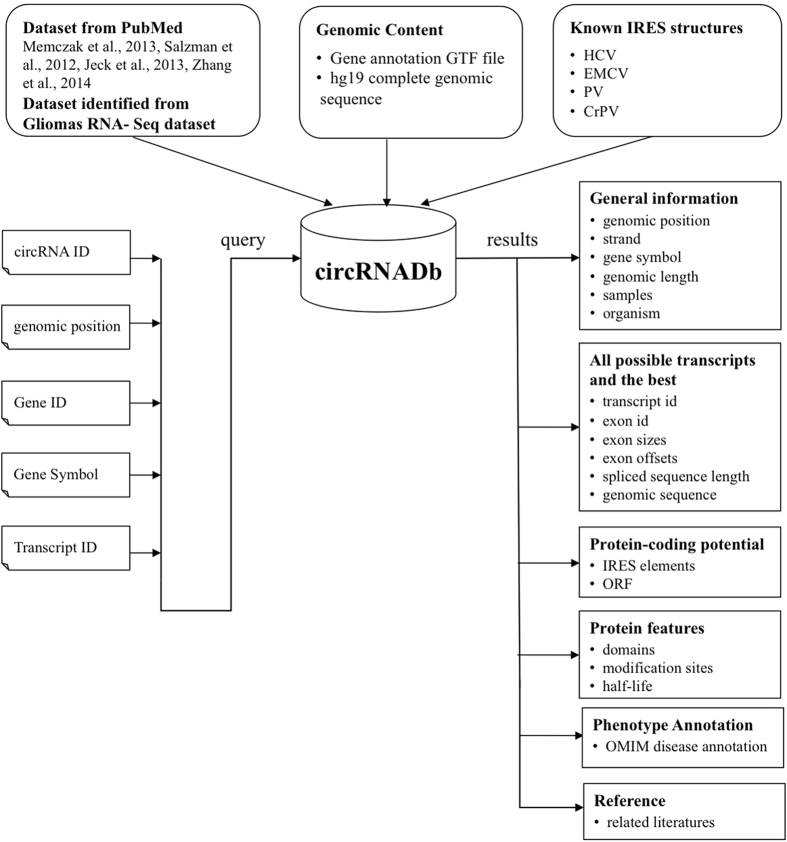
Data sources and the structure of circRNADb.

Finally, the circRNA dataset, together with its protein-coding potential, protein features were merged into circRNADb. The database was configured in the typical WAMP (Windows+Apache+Mysql+PHP) integrated environment, and built by integrating HTML5, CSS3 and Javascript programming languages. The data sources and the structure of circRNADb are shown in Fig. 1.

## Results

As a comprehensive, user-friendly, interactive database, circRNADb provides the following main functions to users, including simple search, advanced search, browse, resource download, and information feedback. It includes 32,914 human exonic circRNAs after filtering for redundancy.

### Search function

There is a search text box in the top right corner of each webpage, users can enter the search terms as required, such as chromosome name, gene symbol, transcript, and other keywords to query the circRNA, then the results that matched the query keywords will be listed in the result page. For example, if the user want to search by gene symbol (e.g., “SDF4”), input the keyword of “SDF4” or “SDF”, circRNADb will return circRNAs from genes matching those keywords (ID: has_circ_21644, chr1:1158623:1159348). The users can also customize searching fields by clicking the “CUSTOMIZE” button. After clicking the circRNA ID in the first column, a web page with detailed information of the circRNA will be loaded.

In addition, users can restrict search terms through advanced search. As many as six fields combined with ‘AND’, ‘OR’ and ‘NOT’ in “Advanced Search” page could be used to retrieve specific circRNAs.

#### Data browse and download

The dataset in circRNADb can be browsed in three options: (1) browse by gene symbol, (2) browse by PubMed ID, and (3) browse by cell type.

In “Browse by Gene Symbol” page, all gene symbols and the total number of circular isoforms produced by parental genes are listed in form of a table. Users can view the detailed information of each parental gene including general information of gene symbol and all circular RNA isoforms by clicking the “Counts” on the right column. In addition, if users want to query a specific gene, the gene symbol or gene ID in NCBI can be used as query terms in the search box for targeted results. This feature allows user to simply and effectively view the information of a parental gene that they are interested in, including all circular transcripts produced.

circRNADb can also be browsed by cell (or tissue) type. In the result page, data are grouped by cell (or tissue) types, which are listed in a table with 11 types of cells and tissues. The total number of circRNAs for each cell or tissue is also listed, and users can click the number to view the detailed list of all the circRNAs identified in the cell or tissue type. This function is quite effective to retrieve all circRNAs expressed in a specific cell or tissue. Finally, all dataset of circRNA in circRNADb can also be downloaded at “Resources” page.

#### Submit a new circRNA and feedback

To make circRNADb more comprehensive and updated, it is important to maintain and update the database. So we designed the submission page for the users to submit their own data to circRNADb and the feedback page for the users to report problems or suggestions in circRNADb. When a user submits a new circular RNA, they need to provide the corresponding information including chromosome name, start position, end position, strand, gene symbol, best transcript, user’s name and email.

#### Detailed information of each circRNA

**Figure 2:**
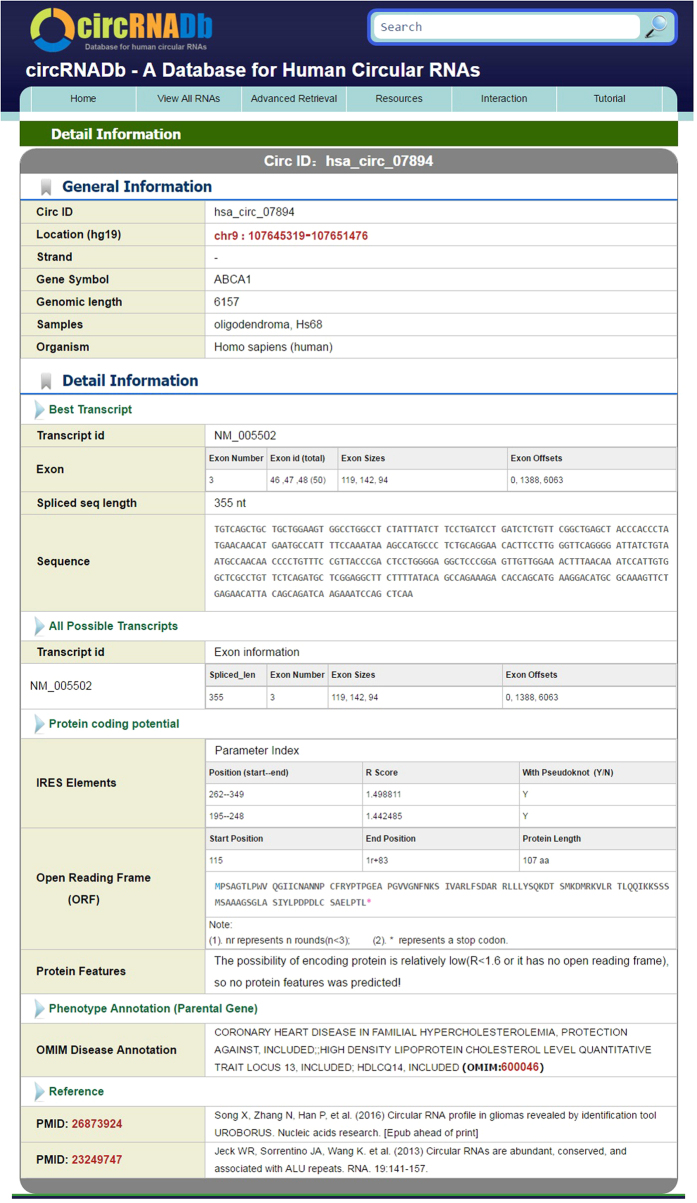
Detailed information web page of a circRNA (ID: hsa_circ_07894).

As mentioned above, users are allowed to view the detailed information of each circRNA by clicking the ID on the first column in the result page. To ensure the accuracy of the database, all the information of each entry was carefully checked manually. As shown in Fig. 2, the page is divided into two major sections: basic information and detailed information. In the basic information section, ID, genomic location, strand, gene symbol, sample name and species of each circRNA are displayed. And the detailed information section provides detailed information for each circRNA.

The detailed information section provides the best matched transcript of the circRNA, its exons information and spliced sequences. According to gene annotation GTF file, all possible circular isoforms and related information are also displayed. Next, in order to study the protein-coding potential of the circRNA, IRES elements and potential ORF longer than 300 bp in each circRNA were predicted. IRES elements with top two highest scores were provided, with details including position, parameter index (R Score and the existence of Pseudoknot). The longest ORF that starts with a start codon (ATG), ends with a stop condon (TAA, TAG or TGA) is shown in the below. There are 11,423 and 16,328 circRNAs predicted to contain IRES element and ORF respectively. 7,010 circRNAs contain both IRES and ORF, approximately accounting for 21.3% of all circRNAs, which are considered as potential protein-coding circRNAs. If the circRNA has the potential to code a protein, protein features including domains, post-translational modification sites and half-life prediction are provided. In addition, we annotated circRNA parental gene associated with human disease (OMIM). At the bottom, literature sources of the circRNA are provided, including PubMed IDs and the detailed references.

The expression evidences were also provided for the circRNA-coding proteins. Two mass spectrometry datasets of human brains from PRIDE database were used to identify proteins exclusively coded by circRNAs. In total, 45 peptides mapped to 46 circRNA-encoding proteins, corresponding to 37 genes, were identified by mass spectrometry. These peptides didn’t belong to any known UniProt human proteins, and were unique to circRNA-encoding proteins. Some peptides were mapped to two different circRNAs, we found that all these are different splicing isoforms from the same gene. Thus, circRNAs from the 37 genes were confidently identified to encode proteins in human brain (Supplementary Table S1). Among the 46 protein coding circRNAs with mass spectrometry evidences at protein expression level, 22 circRNAs were annotated to have IRES element. The representative spectra of one example peptide (“LLQCYPPPEDPAVR”, encoded by has_circ_25375) indicated the high quality of proteomic identification (Supplementary Figure S1). The expression of these circRNA-encoding proteins in human brain indicated that circRNAs might perform functions by translation into proteins.

## Discussions and Conclusions

As a comprehensive human exonic circRNA database with protein-encoding feature annotation, circRNADb is designed to provide a rich data resource for the circRNAs research. circRNADb has collected circRNA dataset from relevant literatures and the brain RNA-seq dataset from our work. In total, we obtained 32,914 non-redundant human exonic circRNAs. circRNADb may facilitate circRNA studies by (1) providing users with detailed genomic information of each circRNA; (2) annotating protein-coding potential of each circRNA; (2) including protein expression evidences of circRNA by mass spectrometry; (3) providing convenient interfaces to retrieve the data.

We will update the newly identified circRNAs, and incorporate their potential functions of encoding proteins or regulating gene expressions. With the improvement of circRNADb, it is expected to become a powerful tool and provide a foundation for further study on circRNA.

## Additional Information

**How to cite this article**: Chen, X. *et al*. circRNADb: A comprehensive database for human circular RNAs with protein-coding annotations. *Sci. Rep.* **6**, 34985; doi: 10.1038/srep34985 (2016).

## Supplementary Material

Supplementary Information
