# Supplementary material for: circRNADb: A comprehensive database for human circular RNAs with protein-coding annotations
Source: Sci Rep. 2016 Oct 11;6:34985. doi: 10.1038/srep34985 (PMC5057092; doi:10.1038/srep34985)
Supplement: Supplementary Information [file srep34985-s1.pdf]

circRNADb：A comprehensive database for human circular RNAs with protein-coding annotations

Xiaoping Chen[1], Ping Han[2], Tao Zhou[3], Xuejiang Guo[3], Xiaofeng Song[1,*],Yan Li[4, *]

1. Department of Biomedical Engineering, Nanjing University of Aeronautics and Astronautics, Nanjing 211106, China;
2. Department of Gynecology and Obstetrics, The First Affiliated Hospital with Nanjing Medical University, Nanjing 210029, China;
3. State Key Laboratory of Reproductive Medicine, Department of Histology and Embryology, Nanjing Medical University, Nanjing 210029, China;
4. Center of Pathology and Clinical Laboratory, Sir Yifu Hospital Affiliated with Nanjing Medical University, Nanjing 211166, China;

*Co-corresponding author,
Email: xfsong@nuaa.edu.cn (X.S.); yanli@njmu.edu.cn (Y.L.)

Supplementary Table S1

Peptide sequence	Peptide length	Missed cleavages	Mass	Charges	PEP	Score	circRNA ID	circRNA length	ORF start site	ORF length	Gene symbol	Dataset (Pride database)	Pubmed	Tissue
MLDAAR	6	0	904.50033	2	0.19315	67.841	hsa_circ_32746	2870	4	2913	AGL	PXD002528	26631761	Temporal lobes (human brain)
ASPCLER	7	0	1060.5538	2	0.06705	71.141	hsa_circ_20832	1859	12	1863	ANK2	PXD002528	26631761	Temporal lobes (human brain)
LLQCYPPPEDPAVR	14	0	1882.9814	2,3	1.8831E-10	125.82	hsa_circ_25375	1847	103	1773	AP2A2	PXD002528	26631761	Temporal lobes (human brain)
EPPGGTGPF	9	0	1086.5549	1	1.1588	0	hsa_circ_04947	452	44	438	BDH1	PXD002528	26631761	Temporal lobes (human brain)
RLETMER	7	1	1162.6331	2	0.022118	93.262	hsa_circ_27654; hsa_circ_00570	2917	14	2970	CIT	PXD002528	26631761	Temporal lobes (human brain)
SQQIQQMADK	10	0	1633.8863	2	0.036179	63.401	hsa_circ_27654; hsa_circ_00570	2917	14	2970	CIT	PXD002528	26631761	Temporal lobes (human brain)
MQQQSTMSWQNLK	13	0	1896.943	3	1.0452	14.03	hsa_circ_04264	805	748	867	DCTN4	PXD000458	26181685	cryptococcal meningitis and normal brains
LYVNQLDHGAR	11	0	1513.8204	3	0.000093249	109.66	hsa_circ_26567; hsa_circ_02306	992	23	987	DTNA	PXD002528	26631761	Temporal lobes (human brain)
RLPEGISASSPVAEEHSLIK	20	1	2577.4532	3,4	4.4502E-15	139.81	hsa_circ_26567; hsa_circ_02306	992	23	987	DTNA	PXD002528	26631761	Temporal lobes (human brain)
MLESSNRLDEEHR	13	1	1843.9049	3	0.025813	61.532	hsa_circ_28404	397	2	405	DTNA	PXD002528	26631761	Temporal lobes (human brain)
TPAEAEMHFLENAK	14	0	2045.0658	3	2.7755E-84	122.45	hsa_circ_02136	1744	224	1551	EPB41L3	PXD002528	26631761	Temporal lobes (human brain)
FDTKPMNLCGR	11	1	1625.8262	3	0.66666	58.37	hsa_circ_02838	710	163	675	ESYT2	PXD000458	26181685	cryptococcal meningitis and normal brains
MGGLANFCYR	10	0	1331.6236	2	1.0778	37.344	hsa_circ_18082	377	341	435	FAF1	PXD000458	26181685	cryptococcal meningitis and normal brains
WRREGLK	7	2	1401.861	2	0.095474	70.912	hsa_circ_08893	461	448	489	FAM126B	PXD002528	26631761	Temporal lobes (human brain)
GTSITGSWTR	10	0	1293.688	2	0.0086677	83.168	hsa_circ_20910	301	150	474	FARSA	PXD002528	26631761	Temporal lobes (human brain)
MLAGPDLK	8	0	1301.7783	3	1.9353	20.902	hsa_circ_11881; hsa_circ_20838	460	95	420	GDAP2	PXD002528	26631761	Temporal lobes (human brain)
IQAAASTPTNATAASGFVR	19	0	1977.0401	2,3	0.70271	57.499	hsa_circ_25280	325	152	582	GSK3B	PXD000458	26181685	cryptococcal meningitis and normal brains
MYLTGMVDK	9	0	1514.8243	2	0.13577	40.002	hsa_circ_16168	938	33	939	GSPT1	PXD002528	26631761	Temporal lobes (human brain)
MLTQPLALMMYMMKK	15	1	2516.4034	2,3,4	0.18475	16.939	hsa_circ_14366	1018	907	1185	KCNQ5	PXD002528	26631761	Temporal lobes (human brain)
EFFDSVR	7	0	1127.5814	2	0.0222	110.34	hsa_circ_26536	904	127	888	LRRFIP1	PXD002528	26631761	Temporal lobes (human brain)
EISDLQETIEWK	12	0	1948.0559	2	0.00000864	165.7	hsa_circ_26536	904	127	888	LRRFIP1	PXD002528	26631761	Temporal lobes (human brain)
TMYNQATQEIAKPSELLTSV R	21	1	2667.4145	3	0.0000595	120.92	hsa_circ_23018; hsa_circ_22823	706	14	720	NCKAP1	PXD000458	26181685	cryptococcal meningitis and normal brains
YNRPVEEWLQEK	12	1	1877.9879	2	8.58E-12	182.71	hsa_circ_10366; hsa_circ_04516	752	381	489	NRXN3	PXD000458	26181685	cryptococcal meningitis and normal brains

YNRPVEEWLQEK	12	1	2048.1097	2,3	2.3994E-06	170.04	hsa_circ_10366; hsa_circ_04516	752	381	489	NRXN3	PXD002528	26631761	Temporal lobes (human brain)
AQPPLSAMMMTLPAHSITSPMGVTLHLLR	29	0	3244.7079	4	0.99488	13.341	hsa_circ_12152	728	117	732	NTRK2	PXD000458	26181685	cryptococcal meningitis and normal brains
DFSWFGFGK	9	0	1377.6961	2	7.1836E-29	134.26	hsa_circ_30879	1960	257	1749	NTRK2	PXD000458	26181685	cryptococcal meningitis and normal brains
PVIFNEPLSFLQR	13	0	1702.9528	2	2.9965E-13	182.71	hsa_circ_17707	416	19	444	OSBPL1A	PXD000458	26181685	cryptococcal meningitis and normal brains
MMIMENGIVSHLK	13	0	1960.0714	2	0.29737	7.0391	hsa_circ_20275	238	223	315	PDIA3	PXD002528	26631761	Temporal lobes (human brain)
GGQICNMMRMMMK	13	1	2044.9941	3	0.21144	2.6344	hsa_circ_29140	716	3	783	POLR2B	PXD002528	26631761	Temporal lobes (human brain)
GEEAVSGLSK	10	0	1433.8132	2	0.18075	54.608	hsa_circ_26646; hsa_circ_29276	1591	1583	1725	POLR3A	PXD002528	26631761	Temporal lobes (human brain)
AHQYEDVVQER	11	0	1516.7392	2,3	0.15189	73.781	hsa_circ_15447	692	33	696	PPP1CB	PXD000458	26181685	cryptococcal meningitis and normal brains
AHQYEDVVQER	11	0	1601.8001	2	0.045684	63.624	hsa_circ_15447	692	33	696	PPP1CB	PXD002528	26631761	Temporal lobes (human brain)
MEQMMIHKTVMMK	13	1	2324.2519	3	0.23318	7.2019	hsa_circ_02497	277	239	330	PTGES3	PXD002528	26631761	Temporal lobes (human brain)
ELDELLK	7	0	1146.674	2	0.17126	76.899	hsa_circ_06367	590	77	528	PTPRD	PXD000458	26181685	cryptococcal meningitis and normal brains
ASLPSESNEPKR	12	1	1771.9834	2	0.16784	45.829	hsa_circ_27515	451	82	447	SEC23IP	PXD002528	26631761	Temporal lobes (human brain)
GQMTDMIHQLLK	12	0	1872.0367	2	0.036099	68.069	hsa_circ_23253; hsa_circ_17819	1241	312	1260	SETD2	PXD002528	26631761	Temporal lobes (human brain)
TLVMLDEQGEQLDR	14	0	1789.9002	2,3	1.4805E-28	252.04	hsa_circ_28122	615	64	576	SNAP25	PXD000458	26181685	cryptococcal meningitis and normal brains
TLVMLDEQGEQLDR	14	0	1874.9611	2	0.00091107	81.278	hsa_circ_28122	615	64	576	SNAP25	PXD002528	26631761	Temporal lobes (human brain)
TLVMLDEQGEQLDRVEEGMNHINQDMK	27	1	3629.7893	3,4	7.7138E-50	184.45	hsa_circ_28122	615	64	576	SNAP25	PXD002528	26631761	Temporal lobes (human brain)
VEEGMNHINQDMK	13	0	1831.88	3	6.8191E-13	182.07	hsa_circ_28122	615	64	576	SNAP25	PXD000458	26181685	cryptococcal meningitis and normal brains
MCMISLR	7	0	1053.5255	2	0.59013	39.542	hsa_circ_16032	1624	1563	1737	SNX13	PXD000458	26181685	cryptococcal meningitis and normal brains
DPTNIQLSK	9	0	1472.8604	2,3	0.00028304	132.91	hsa_circ_02276	1102	1098	1158	SPTAN1	PXD002528	26631761	Temporal lobes (human brain)
LQTASDESYKDPTNIQLSK	19	1	2569.3601	3	8.0392E-46	274.81	hsa_circ_02276	1102	1098	1158	SPTAN1	PXD000458	26181685	cryptococcal meningitis and normal brains
LQTASDESYKDPTNIQLSK	19	1	2824.5427	2,3,4	7.7405E-43	203.89	hsa_circ_02276	1102	1098	1158	SPTAN1	PXD002528	26631761	Temporal lobes (human brain)
ICCLSKMMYTTCPDALFNELVK	22	1	3380.7283	3	0.11939	35.389	hsa_circ_00115	469	436	525	STXBP1	PXD002528	26631761	Temporal lobes (human brain)

MLHYLVLVTGCMSVGK	16	0	2095.1236	4	1.0895	6.7282	hsa_circ_08055	665	96	636	SYNJ1	PXD000458	26181685	cryptococcal meningitis and normal brains
MIPSFMTMDQSRK	13	1	2029.0565	3	0.18407	13.404	hsa_circ_06318	751	73	708	TUBGCP3	PXD002528	26631761	Temporal lobes (human brain)
QHQFQFLLK	9	0	1645.971	2	0.03857	72.547	hsa_circ_08565	944	889	1065	ULK2	PXD002528	26631761	Temporal lobes (human brain)
TIHFLLI	7	0	1084.6848	2	4.8488	0	hsa_circ_13000	959	73	954	UPF2	PXD002528	26631761	Temporal lobes (human brain)

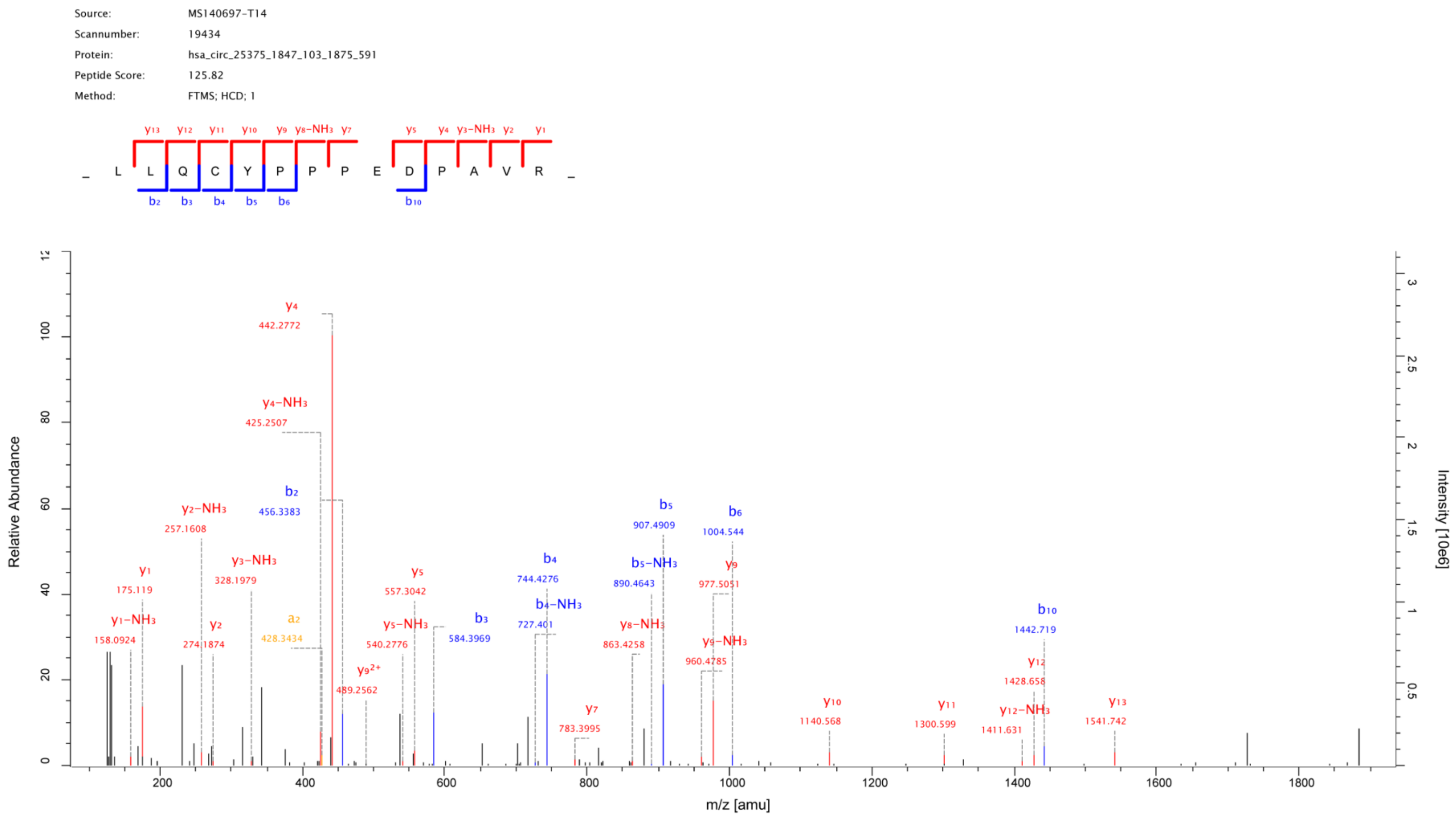

Figure S1 The representative spectra of one example peptide ("LLQCYPPPEDPAVR", encoded by has_circ_25375)
